# Preparation and Properties of Brake Friction Materials Reinforced with Coconut Fiber and Dypsis Lutescens Fiber

**DOI:** 10.3390/ma17163926

**Published:** 2024-08-07

**Authors:** Chaotian Wang, Ruimin Li, Huidong Lin, Shengwang Yuan, Lining Wang, Yunhai Ma

**Affiliations:** 1 Key Laboratory of Bionic Engineering (Ministry of Education), College of Biological and Agricultural Engineering, Jilin University, Changchun 130022, China; ztwang22@mails.jlu.edu.cn (C.W.); rmli23@mails.jlu.edu.cn (R.L.); linhuidong97@163.com (H.L.); ysw5968@163.com (S.Y.); wangln@jlu.edu.cn (L.W.); 2 Institute of Structured and Architected Materials, Liaoning Academy of Materials, Shenyang 110167, China

**Keywords:** plant fiber, brake friction materials, tribological properties, formulation optimization

## Abstract

Brake friction material reinforced with coconut fiber and dypsis lutescens fiber was designed and prepared in this study. Specimens incorporating 0–8 wt.% of coconut fibers or dypsis lutescens fibers were fabricated. The effect of the content of these reinforcing fibers on the overall properties of brake friction materials was systematically investigated. The results indicate that the inclusion of reinforcing fibers in the formulation of brake friction materials can improve the physical properties and friction and wear properties of brake friction materials. The specimen incorporating 6 wt.% plant fiber obtained the optimal comprehensive performance with excellent fade resistance and recovery properties, and better wear resistance. In order to further investigate their performance, nine hybrid fiber brake friction materials were designed using the golden section method and orthogonal test method. The study indicated that the F-6 hybrid fiber-reinforced brake friction materials have better physical properties, thermal degradation resistance, recovery properties, and abrasion resistance than the single-fiber-reinforced brake friction materials. This study provides new concepts for the preparation of fiber-reinforced brake friction materials as well as formulation optimization.

## 1. Introduction

In contemporary society, friction materials, as a key functional material, play an indispensable role in several fields, especially in transportation, agriculture, military, and engineering [1,2,3]. These materials have a direct impact on the safety, stability, and reliability of mechanical devices and are the basis for ensuring the smooth operation of modern mechanical systems [4,5]. Brake friction material reinforced with natural plant fibers is an innovative and environmentally friendly material [6]. The use of natural plant fibers as reinforcing fibers not only significantly improves the performance of brake friction materials, but also reduces production costs and enhances environmental friendliness [7,8].

Numerous studies have demonstrated that natural plant fibers have a positive impact on various aspects of brake friction material performance [9]. Kchaou M et al. studied manzanita fibers and designed manzanita fiber-reinforced composite friction materials, comprehensively characterizing their fiber orientation and distribution. The study found that the mechanical properties and thermal stability of the composite friction material were significantly improved with the addition of 10 wt.% manzanita fibers, which had good friction factor and wear-resistant properties [10]. Kumar N et al. prepared a new environmentally friendly brake friction material using pine leaf fibers [11]. Tests revealed that the pine leaf fibers have good mechanical properties and thermal stability. The study pointed out that the unique structure and composition of the pine leaf fibers make them compatible with the base material [11,12]. However, the feasibility of using plant fibers in brake friction materials has not been fully explored, especially in the case of plants with high fiber content and good physical properties, such as the sunflower and coconut, which have not been studied in sufficient depth [13,14].

The chemical composition of coconut fiber is 36% to 43% cellulose and 41% to 45% lignin [15]. The cellulose content of Sanwei Kui fiber is approximately 51.11 wt%, the lignin content is about 23.71 wt%, and the density is about 1.35 g/cm^3^ [16]. Cellulose is the primary chemical component responsible for fiber stability and strength. Treatment with an alkali-catalyzed process has been found to produce excellent total lignin concentrations and a higher cellulose fraction [17,18]. These fibers are used as friction materials due to their high tensile strength and low elastic modulus compared to other fibers, which can significantly improve the wear resistance and lifespan of materials [19]. Additionally, their low density provides lightweight material options. They are natural, renewable, and easily biodegradable, reducing the environmental burden and being cost-effective.

Braking friction materials are multi-component composites primarily consisting of reinforcing fibers, binders, fillers, and friction performance regulators. The performance of braking friction materials will also be affected by the different proportions of each component and the raw materials [1]. Aiming at the above research background, this study starts with two plants, coconut and dypsis lutescens, respectively, to study the feasibility of these fibers as reinforcement for automotive brake friction materials and to equip hybrid plant fiber friction materials through the golden section method and orthogonal test method [20]. The golden section method is an optimization technique to find the best value in a one-dimensional space. Correlating the golden section sequence with the mass fraction of each component in the friction material formulation enables optimal design and calculation [21]. The orthogonal design of experiments is a practical method for designing and optimizing multifactorial and multilevel experiments, which can improve experimental efficiency and reduce experimental costs. Brake friction materials with nine different ratios of coconut fibers blended with dypsis lutescens fibers were prepared based on the golden section sequence approach, drawing on the orthogonal design of the experiment methodology [22]. These friction materials were tested for physical properties and friction and wear properties, and their tribological properties were compared and analyzed. Then the optimal formulations were selected by the fuzzy comprehensive evaluation method. These were compared and analyzed against the brake friction materials with optimal performance prepared using a single fiber, verifying the reasonableness of the method [23]. Finally, nine brake friction materials were subjected to surface micromorphology analysis to study their wear mechanisms [1,24].

## 2. Materials and Methods

### 2.1. Raw Materials

Two different plant fibers, i.e., coconut fiber or dypsis lutescens fiber, were used as reinforcing plant fibers, together with composite mineral fibers to form the reinforcing fiber components. The phenolic resin was used as the binder. Calcium carbonate, vermiculite powder, and precipitated barium sulfate were used as fillers [25,26]. Flake graphite, petroleum coke, alumina oxide, zinc stearate, and friction dust were applied as friction performance modifiers [27]. The specifications and manufacturer information of the above raw materials are shown in Table 1. We have rationalized the ratio of each group and designed the formulation of brake friction materials [28]. Table 2 and Table 3 show the specific formulations for this test. Since the content of precipitated barium sulfate has a small effect on the performance of friction materials, the precipitated barium sulfate is used as the corresponding variable, and the ratios of coconut fiber and dypsis lutescens fiber are varied to investigate the effect of formulations with different fiber contents on the performance of brake friction materials [12,20,29].

Then, a mixture of coconut fiber, dypsis lutescens fiber, and composite mineral fiber was used, with their contents set at 7.64 wt.%, 12.36 wt.%, and 20 wt.%, respectively, using the golden section method. Using the orthogonal test method, the three types of fibers—coconut fiber, dypsis lutescens fiber, and mineral fiber—were set as three factors, and three levels were set for each factor [30]. The content and information of the raw materials are shown in Table 2 and Table 3, respectively. The design optimization resulted in nine brake friction material formulations, as shown in Table 4 [31,32].

### 2.2. Fabrication of Specimen

**Figure 1:**
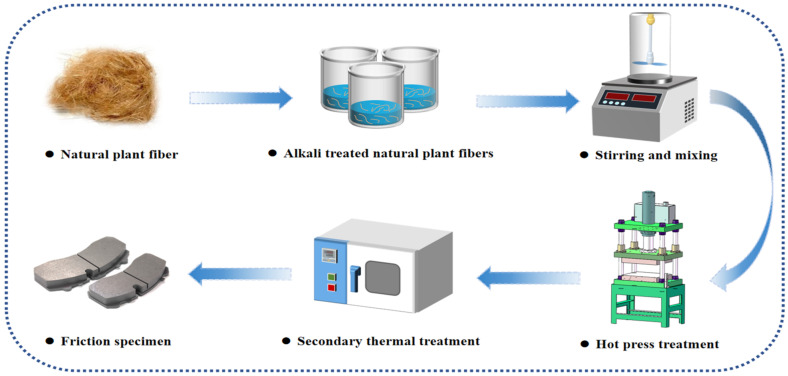
The process of making friction specimens.

The main preparation steps of reinforced brake friction materials are alkali treatment of cut plant fibers, batching and blending, hot pressing, and heat treatment. The fabrication process is shown in Figure 1.

First, the plant fibers were fully soaked in water for 24 h, after which a pulverizer was used to pulverize the fibers so that the fibers were exposed. The fibers were subsequently dried in a constant-temperature oven until they reached a constant weight and then screened with a sampling sieve to obtain 40–60 mesh short-cut fibers. Then, the cut fibers were immersed in a NaOH solution with a mass fraction of 1%, and the ratio of the fiber mass to the volume of the solution was 1:30 g/mL. They were fully immersed for 30 min and stirred continuously with a glass rod during the immersion process. At the end of the reaction, the alkali-treated fibers were continuously rinsed with distilled water until the pH of the rinsing solution was neutral. Then, the fibers were placed in a drying oven and dried at 70 °C until constant weight, to obtain the samples of alkali-treated plant fiber.

Next, the formulations of brake friction materials shown in Table 2 and Table 3 were weighed proportionally. A batch stepwise dosing method [29] was adopted to mix the weighed raw materials thoroughly using a plow and harrow mixer (JF810, Wangda, Changchun, China). The pre-weighed fibers of each component were placed into the mixer and mixing and stirring were carried out, setting the main shaft speed at 700 rpm, the churning knife speed at 1415 rpm, and mixing for 5 min to obtain a relatively loose and non-agglomerated fiber material. Pour in phenolic resin, friction performance regulator, and other fillers to mix materials, set the spindle speed of 1400 rpm, stirring knife speed of 2830 rpm, and mix for 10 min to obtain a uniform fiber powder material. Intermittent mixing methods were used throughout the procedure, which involves mixing for 30 s and pausing for 10 s until the full mixing process is completed [33].

In the third step, the material obtained after homogeneous mixing is put into the mold cavity of the special hot press (JFY40, Wangda, Changchun, China) for hot press molding treatment, with a pressing pressure of 11 MPa and a pressing temperature of 170 °C. Pressure preservation occurred 6 times in total, with the 1st and 2nd pressure preservation times being 5 s, the 3rd, 4th, and 5th pressure preservation times being 10 s, and the 6th pressure preservation time being 900 s. Exhausting was performed 6 times, with the 1st to the 5th exhausting times being 5 s, and the 6th exhausting time being 30 s. Before the start of the test, the mold cavity is preheated to over 150 °C. The material is quickly poured into it for the test to prevent the denaturation of the raw material from adversely affecting the performance of the friction material before the hot pressing test [1,34].

**Figure 2:**
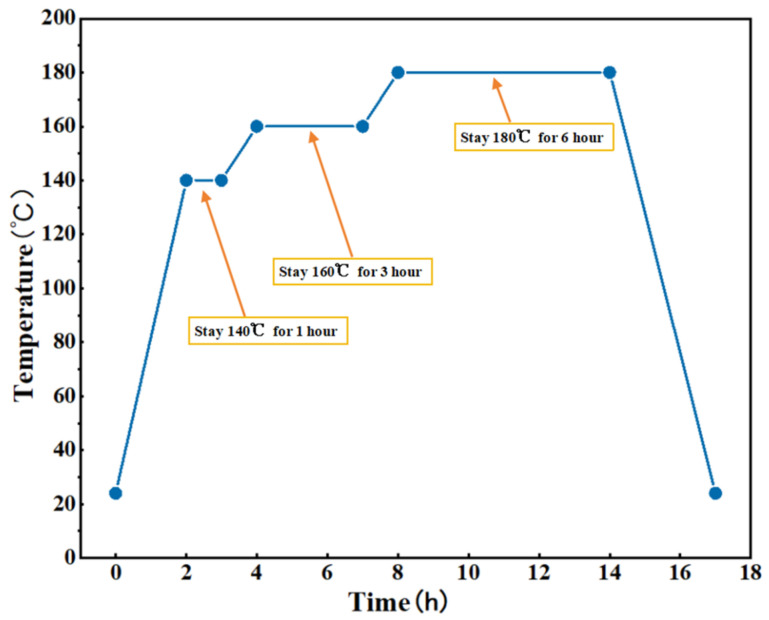
Heat treatment process diagram.

After hot pressing, a heat treatment oven (JF980S, Wangda, Changchun, China) is used to heat treat the pressed product to eliminate residual stress and thermal stress. The heat treatment process is shown in Figure 2.

### 2.3. Testing Methods and Equipment

The density of the specimen was tested using the Archimedes drainage principle, the hardness test was conducted using a Rockwell hardness tester (TH301, Shidai, Beijing, China), and the impact strength test was conducted using a pendulum impact tester (XJ-40A, Wuzhong, China). The test method is as follows: first, the brake friction material is cut into a size of 55 mm × 6 mm × 4 mm and placed on a table. Then, a pendulum suspended at a high altitude swings freely and breaks the processed sample. The energy absorbed at this time can be calculated by Formula (1). Each sample is tested 5 times and the average value is taken.

(1) $$a_{k}=A_{k}/\left(b\times d\right)$$

where $a_{k}$ was the impact strength of the tested specimen (J/cm^2^); $A_{k}$ was the impact energy consumed to break the specimen (J); b was the width of the specimen (cm); and d was the thickness of the specimen (cm).

**Figure 3:**
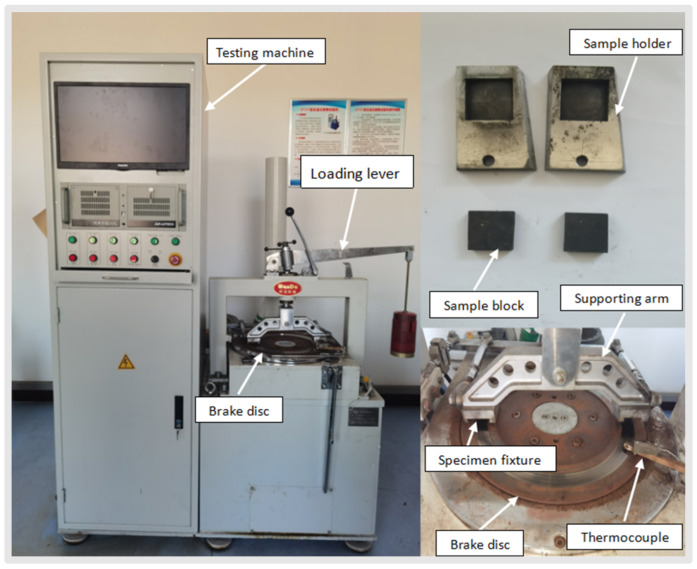
JF151 fixed speed type friction tester.

The tribological performance of specimens was evaluated by a constant-speed tester (JF150F-II, Wangda, Changchun, China, shown in Figure 3) according to GB/T 5763-2008 [35]. Specific test methods are as follows: first of all, the brake friction material was mechanically cut into 25 mm × 25 mm × 6 mm size and this specimen was fixed on the friction disk. Then, the specimen was heated using a heating tube before grinding. It must be taken into account that the temperature should always be lower than 100 °C, while the contact surface of the specimen with the friction disc should be more than 95%. At the end of the break-in phase, the specimen was cooled to room temperature, and its thickness was measured in 5 places (4 corner points and 1 center point). After this, the temperature of the friction disc was increased to 350 °C After reaching the maximum temperature, the friction disc was cooled so that its temperature dropped at intervals of 50 °C, and the friction value was determined during 1500 revolutions of the disc. Friction measurement was repeated at a temperature of 100 °C for 500 revolutions of the friction disk. Each brake friction material was tested five times, and the average friction value was calculated [36,37].

The friction factor and wear rate at each test temperature were calculated by Equations (2) and (3), respectively [38]:

(2) $$\mu =f/F_{N}$$

(3) $$\Delta V=\frac{1}{2\cdot \pi \cdot r}\cdot \frac{A\left(d_{1}-d_{2}\right)}{N\cdot f}$$

where f was the friction force between the specimen and brake disc (*N*); $F_{N}$ was the force generated by normal pressure on the surface (N); r was the distance from the specimen center to the brake disc center (r = 0.15 m);***N*** was the number of revolutions (*N* = 5000); A was the contact surface area, which was 625 mm^2^; and $d_{1}$ and $d_{2}$ were the initial and final thickness of the specimen (mm), respectively.

The recovery and recession properties of brake friction materials were calculated using Formulas (4) and (5) [39] as follows:

(4) $$R=\frac{\mu_{R_{100}}}{\mu_{F_{100}}}\times 100\%$$

(5) $$F=\frac{\mu_{F_{100}}-\mu_{F_{350}}}{\mu_{F_{100}}}\times 100\%$$

where $\mu_{R_{100}}$ was the brake friction material sample at 100 °C during the recovery test, $\mu_{F_{100}}$ was the brake friction material sample at 100 °C during the fade test, and $\mu_{F_{350}}$ was the brake friction material sample at 350 °C during the fade test.

After the friction-wear test, the wear surface micromorphology of brake friction material specimens with different fibers and fiber contents was analyzed using scanning electron microscopy (SEM, EVO-18, ZEISS, Jena, Germany).

## 3. Results and Discussion

### 3.1. Performance Analysis of Single-Plant Reinforced Fibers

#### 3.1.1. Physical Properties

**Figure 4:**
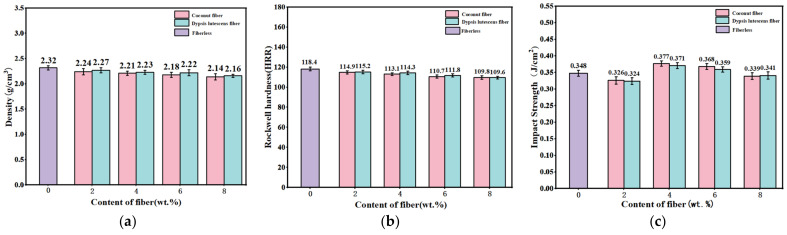
(**a**) The density of added single-fiber specimens; (**b**) the hardness of added single-fiber specimens; (**c**) the impact resistance of added single-fiber specimens.

Figure 4a presents the density test results of coconut fiber and dypsis lutescens fiber-reinforced brake friction material. It can be seen that the overall density of the brake friction material gradually decreases with the increase in fiber content. This is primarily because the density of the reinforcing fibers is lower than that of precipitated barium sulfate [40]. In addition, the density of the specimens containing the dypsis lutescens fiber is higher than that of the coconut fiber specimens, which is mainly due to the higher cellulose content of the dypsis lutescens fiber than that of the coconut fibers after the alkali treatment, which manifests itself as an increase in the density of the friction material. Due to the limited mass fraction of reinforcing fibers in the specimens, the effect on the density of the brake friction material was small [41].

The results of the hardness test of brake friction materials reinforced with plant fibers are given in Figure 4b. The hardness and density of the two fiber-reinforced specimens have the same trend (gradually decreasing) as the fiber content increases, and the hardness of the specimen with coconut fibers is slightly less than that of the specimen with dypsis lutescens fibers. The hardness of the friction material was highest, at 118.4 HRR, when this material did not contain plant fibers. When the fiber content in the friction material was reduced to 8 wt.%, the hardness decreased to a minimum value of 109.6–109.8 HRR. This is mainly due to the higher hardness of precipitated barium sulfate in the friction material than the reinforcing fibers. In addition, the hardness values of all the specimens were within reasonable limits and would not have a significant effect on the performance of the brake friction materials [42].

Impact strength is the impact resistance of friction materials and can be used to evaluate their toughness and brittleness [43]. Figure 4c presents the results of the impact strength test of coconut fiber and dypsis lutescens fiber-reinforced brake friction material. The impact strength of the specimens with different fiber content is within a reasonable range, and there is no obvious pattern of change. The impact strength of samples reinforced with fibers of dypsis lutescens was slightly less than that of samples reinforced with coconut fibers [44]. The impact strength of the brake friction material is higher when the content of plant fibers is 4–6 wt.%. This can be explained by the fact that the average fiber content has a higher strength of their interfacial connection with the collective interface, which allows stress to be transferred more efficiently during impact loading, thereby exhibiting higher impact strength [45,46].

#### 3.1.2. Tribological Properties

**Figure 5:**
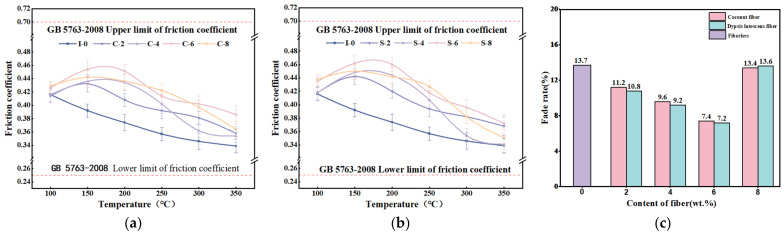
Variation curve of friction factor of specimen: (**a**) coconut fiber reinforced friction material; (**b**) dypsis lutescens fiber reinforced friction material; (**c**) fade rate of added single-fiber specimens.

The results of the decline test are shown in Figure 5a,b. The friction factor of the specimens without added reinforcing fibers decreases with increasing temperature, while the brake friction material with added fibers shows a tendency to increase and then decrease. In the range of 100 °C–150 °C, the friction factor of the specimens containing reinforcing fibers increases slightly, and its value decreases gradually with a further increase in temperature. This is because fibers and some hard particles exposed on the friction surface in the range of 100 °C to 150 °C increase the friction between the friction disc and the friction material, which is manifested as an increase in the friction factor [47]. When the temperature is increased from 150 °C to 350 °C, coconut fibers, dypsis lutescens fibers, and the phenolic resin matrix undergo carbonization, which decreases the friction factor with its lubricating effect [48]. Furthermore, comparing Figure 5a,b, it can be seen that the friction factor at the initial stage of the test (from 100 to 150 °C) for the brake friction material reinforced with dypsis lutescens fibers is slightly larger than that for the brake friction material reinforced with coconut fibers. As the temperature increases from 150 °C to 350 °C, the friction factor of the specimen reinforced with dypsis lutescens fibers decreases slightly more than that of the specimen reinforced with coconut fibers. This may be because the cellulose content of the dypsis lutescens fibers is greater than that of the coconut fibers, and the effect of cellulose friction with the friction discs is more pronounced when the temperature is low. However, as the temperature rises, its carbonization increases, resulting in a reduction in the friction factor [49].

Figure 5c gives the results of the fade rate test, from which it can be seen that the friction factor of all specimens is in line with the provisions of the national standard GB 5763-2008 [35]. The fade rate of the specimens reinforced with coconut fibers showed consistent trends to increase concerning the specimens reinforced with dypsis lutescens fibers. The results showed that the brake friction material with 6 wt.% fiber content has the best performance of heat fade resistance. The fade rate of specimens reinforced with 6 wt.% plant fibers was minimal (7.2–7.4%), indicating that fiber addition enhances the resistance of brake friction materials to thermal fading. [50].

**Figure 6:**
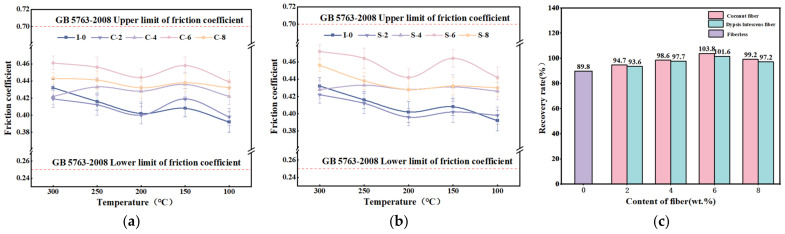
Recovery curves of friction factor of specimens: (**a**) coconut fiber reinforced friction material; (**b**) dypsis lutescens fiber reinforced friction material; (**c**) recovery rate of added single-fiber specimens.

The recovery test results are shown in Figure 6a,b. The friction factor of each specimen exhibits a similar trend. As the temperature decreases from 300 °C to 100 °C, the friction factor of each specimen initially decreases, then increases, and then decreases. Among them, the friction factors of C-6 and S-6 specimens are the highest, with the dypsis lutescens fiber-reinforced specimens showing slightly greater friction factor variation compared to the coconut fiber-reinforced specimens. When the temperature was reduced from 300 °C to 250 °C, the friction factor of the dypsis lutescens fiber-reinforced specimens was slightly larger than that of the coconut fiber-reinforced specimens. However, as the temperature continued to decrease (250 °C to 100 °C), the friction factor of the coconut fiber-reinforced specimens was larger than that of the dypsis lutescens fiber-reinforced specimens [49]. During the cool-down test phase, the friction factors of both brake friction materials were relatively stable and within reasonable limits, although the fluctuation of the friction factor of the dypsis lutescens fiber-reinforced specimens was larger than that of the coconut fiber-reinforced specimens.

The recovery rate test results are shown in Figure 6c. It can be seen that the recovery rates for reinforced specimens with equal content of plant fibers are almost the same. The specimens containing 6 wt.% plant fibers had the greatest recovery (101.6% and 103.8%), while the unreinforced specimen had the least recovery (89.8%). This indicates that the addition of plant fibers can enhance the recovery performance of the brake friction material, which is improved compared with that of the brake friction material without added fiber. With the increase in the fiber content, its recovery rate increases, but beyond a certain level, it will have a certain negative impact on its friction performance [51].

**Figure 7:**
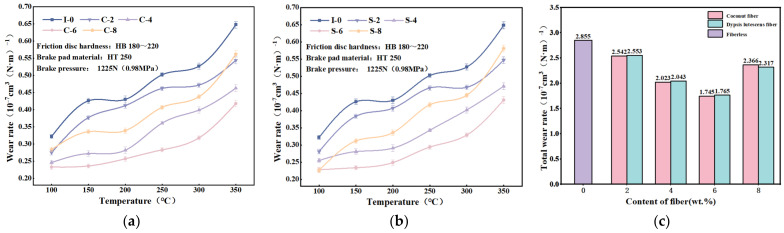
Specimen wear rate change curve: (**a**) coconut fiber reinforced friction material; (**b**) dypsis lutescens fiber reinforced friction material; (**c**) total abrasion rate of added single-fiber specimens.

Figure 7a,b present the curve of wear rate versus temperature for each specimen. The figures indicate that the wear rate of brake friction materials is significantly influenced by temperature and increases as the temperature rises. [52,53]. This may be due to the phenomenon of thermal decomposition of phenolic resin at elevated temperatures, resulting in a weakening of the bond between the components in the friction material and an increase in its wear. The abrasion rate of the dypsis lutescens fiber-reinforced specimens was slightly larger than that of the coconut fiber-reinforced specimens, and the trend of the abrasion rate of the dypsis lutescens fiber-reinforced specimens was more pronounced with the increase in the fiber content. The wear rate of I-0 brake friction material specimens was greater than that of the brake friction material specimens with other fiber contents at all temperatures, indicating that the addition of coconut fibers or dypsis lutescens fibers can improve the wear resistance of brake friction materials [54].

The total wear rate of each specimen at different temperatures is shown in Figure 7c. The total wear rate of the brake friction material shows a tendency to decrease and then increase with the increase in fiber content. The specimens with 6 wt.% addition of plant fibers had the smallest wear rate. As a result of the addition of 6 wt.% fibers, the wear rate of the brake friction materials can be reduced by 38.2%. Therefore, the addition of plant fibers improves the wear resistance of these materials.

#### 3.1.3. Friction Wear Mechanism

Primary and secondary plateaus, adhesion pits, wear debris, and grooves on wear surfaces can be used to analyze the wear mechanism of friction materials [55]. Scanning electron microscopy was used to characterize the wear surface morphology and to analyze the wear mechanism to derive the mechanism of improved wear resistance of brake friction material specimens with different fibers and different fiber contents [56].

**Figure 8:**
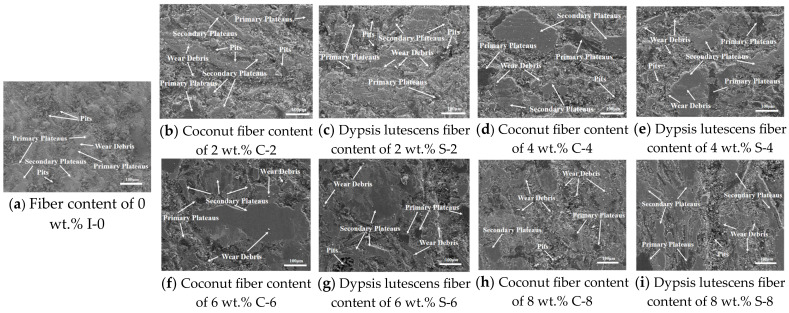
Wear surface micromorphology of single-fiber-added specimens.

As can be seen from Figure 8a, a large number of primary contact plateaus, abrasive chips, spalling pits, and loose substrates appeared on the wear surface of the I-0 specimen without added fibers, which indicates that the bonds between the specimen components are poor and the wear rate is high. A large number of hard chips and particles (alumina, etc.) will be generated by high-temperature and high-pressure impacts. These byproducts act as an abrasive when the brake disc rubs against the brake friction material. These by-products cause scraping and collisions at the interface, leading to material shedding and the formation of numerous spalling pits. Under the action of friction, the friction interface produces some bonding points, which are later cut off as friction proceeds by shear forces and other influences, resulting in increased wear [54,57]. From the above study, it is concluded that the main forms of wear of I-0 brake friction material without added reinforcing fibers are three-body abrasive wear and adhesive wear [41].

Compared with the I-0 specimens without added fibers, the wear surfaces of the other brake friction material specimens were relatively smooth, exhibiting fewer flaking pits and wear debris. This improvement is primarily attributed to the addition of plant fibers, which enhanced the bonding between the components and produced a mechanical self-locking with the base material, resulting in the improvement of the brake friction material’s strength and tribological properties [58]. A comparison of Figure 8b–g indicates that the wear of the brake friction material specimens gradually decreases as the fiber content increases. The wear surfaces of the C-6 and S-6 brake friction material specimens were the smoothest, with more secondary contact plateaus and relatively few abrasive chips and hard particles, which is consistent with the lowest wear rate data for this brake friction material. However, when the fiber content was too high (Figure 8h,i), a certain amount of abrasive debris and hard particles appeared, and scratches and spalling pits appeared along the friction direction. This is primarily because the high content of fibers reduces the bonding effect of the phenolic resin binder, which reduces the strength of the entire brake friction material, and is susceptible to fracture or detachment at high temperatures and under the action of shear forces [51,59].

### 3.2. Performance Analysis of Blended Plant Fiber

#### 3.2.1. Physical Properties

**Figure 9:**
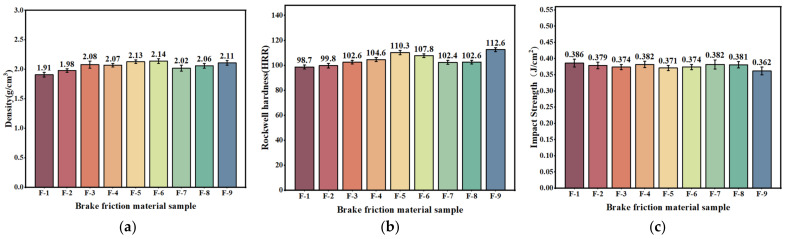
(**a**) The density of friction specimens with mixed plant fibers; (**b**) the hardness of friction specimens with mixed plant fibers; (**c**) the impact resistance of friction specimens with mixed plant fibers.

The density test results of the hybrid fiber reinforced brake friction material specimens prepared using nine formulations designed by the golden section method and orthogonal test method are shown in Figure 9a. Specimen F-6 has the highest density of 2.14 g/cm^3^. The densities of all the brake friction material specimens are within a reasonable range (1.6–2.3 g/cm^3^). The F-1 brake friction material specimen has the lowest density of 1.91 g/cm^3^. This is because the F-1 brake friction material specimen has more fiber content compared to other brake friction material specimens and its density decreases as the plant fiber content increases [60]. The density of the brake friction material specimens prepared in this section slightly decreases due to the increase in fiber content compared to the brake friction material specimens in the previous section [41,61].

The trend of changes in the hardness of brake samples reinforced with hybrid fibers is similar to the density change (Figure 9b). With the increase in fiber content, the hardness of the brake friction material specimen gradually decreases, but the overall change is not large [62]. Compared with the hardness test results of the brake friction material specimens in the previous section, the hardness of the brake friction material specimens in this section is relatively reduced.

With the increase in fiber content, the impact strength of the brake friction material specimens did not show a clear pattern of change (Figure 9c). The impact strength of the F-1 brake friction material specimen is the largest, 0.386 J/cm^2^, and the impact strength of the F-9 brake friction material specimen is the smallest, 0.362 J/cm^2^. The impact strength of all brake friction material specimens is following GB/T 5763-2008 [35]. Meanwhile, compared with the results of the previous section of the brake friction material specimen impact strength test, this section of the brake friction material specimen impact strength is relatively increased [44].

#### 3.2.2. Tribological Properties

**Figure 10:**
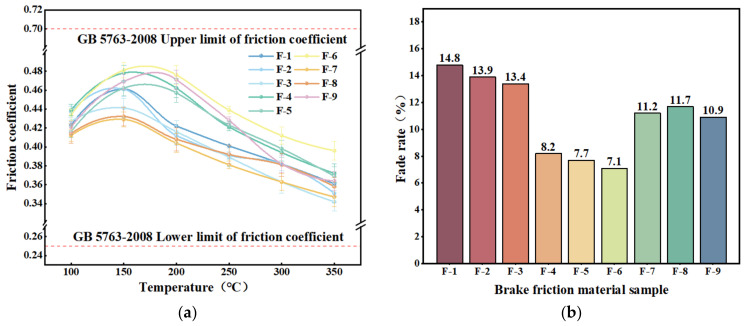
Fade test results of friction specimens with mixed plant fibers: (**a**) plot of friction factor change during fade test stage; (**b**) fade rate.

A graph of the friction factor variation with temperature for nine specimens of hybrid fiber-reinforced brake friction material during the fading (warming) test phase is presented in Figure 10. It is evident from Figure 10a that as the test temperature rises, the specimens’ friction factor first increases and subsequently decreases. The test results of the brake friction material specimens supplemented with dypsis lutescens and coconut fibers in the preceding section are comparable to this trend. The fiber content increased during the test, improving the scraping action between the brake friction material specimen and the friction disc, which caused the increase in friction factor. Initially, the friction disc and the brake friction material specimen did not match optimally. The subsequent reduction in the friction factor may be due to the thermal decomposition of the phenolic resin binder and the carbonization of the plant fibers in the brake friction material specimens during high-temperature operation, resulting in some lubrication of the friction surfaces [59,63]. Compared to other brake friction material specimens, the F-6 specimen has the largest friction factor and the least amount of temperature variation in its friction factor. The F-6 brake friction material specimen has a higher fading friction factor when compared to the specimen with the best heat degradation performance in the preceding section. Overall, the fade friction factors of the nine brake friction material specimens conform to the national standard GB 5763-2008 [35].

The fade rate of the brake friction material specimens was calculated according to Equation (5), as shown in Figure 10b. The specimen of brake friction material with the lowest degradation rate, F-6, is 7.1%, whereas the specimen with the highest degradation rate, F-1, is 14.8%. This could be due to F-1 having a comparatively high plant fiber content. When friction occurs, more plant fibers are exposed to the friction surface, preventing stable tribological performance. This phenomenon is more noticeable with higher plant fiber concentrations [49,50].

**Figure 11:**
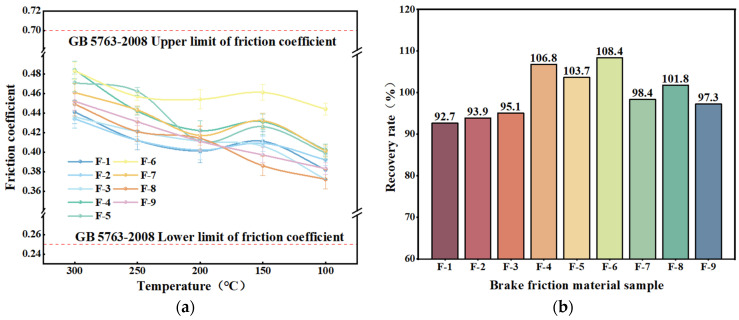
Recovery test results of friction specimens with mixed plant fibers: (**a**) plot of friction factor change during recovery test phase; (**b**) recovery rate.

The variation of friction factor with decreasing temperature for the hybrid fiber reinforced brake friction material specimens during the recovery (cool down) test phase is shown in Figure 11a. During the recovery test, the trend of the friction factor of the specimens was basically similar. The friction factor gradually decreased when the test temperature decreased from 300 °C to 200–150 °C. The specimens’ friction factor marginally increased when the test temperature dropped to around 150 °C and subsequently decreased as the temperature dropped further. The increase in the friction factor is mainly due to the generation of hard particles at the friction interface between the specimen and the friction disk within this temperature range. These particles continuously abrade the braking friction material substrate and the friction disk. The decrease in the friction factor is related to the generation of abrasive debris and changes in the mobility of this debris at the friction interface [56]. The F-6 specimen has the largest friction factor and exhibits relatively small fluctuation with temperature changes compared to other specimens. Additionally, its recovery friction factor is generally higher than that of the brake friction materials with the best recovery performance discussed in the previous section [49]. In general, the restoration friction factors of the nine brake friction materials meet the requirements of GB 5763-2008 [35].

The recovery rates of the brake friction material specimens were calculated according to Equation (4) as shown in Figure 11b. The recoveries of the hybrid fiber-reinforced brake friction material specimens are in the following order from largest to smallest: F-6 > F-4 > F-5 > F-8 > F-7 > F-9 > F-3 > F-2 > F-1. The results showed that the F-6 hybrid fiber-reinforced brake friction material specimen has the best recovery performance. [51].

**Figure 12:**
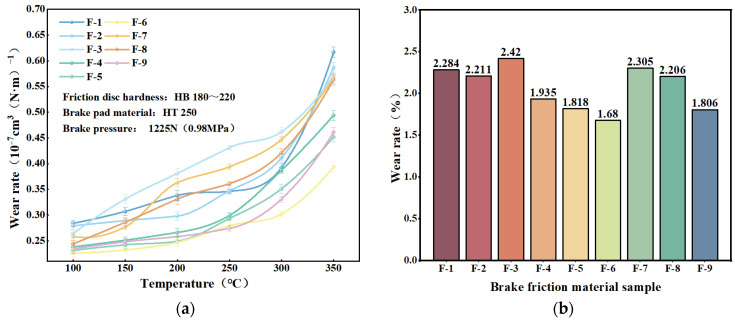
The wear rate of friction specimens with mixed plant fibers: (**a**) wear rate variation curve; (**b**) total wear rate.

The results of the wear rate tests for the nine specimens are shown in Figure 12. The wear rate of all brake friction material specimens increased with rising test temperatures, which is similar to the wear rate tests of single plant reinforced fiber specimens discussed in the previous section [64,65]. At the beginning of the test phase (100–200 °C), the temperature had not yet reached the decomposition temperature of organic materials such as phenolic resins. Consequently, the bonding between the component raw materials remained relatively strong, and the wear rate of the brake friction material specimens increased slowly [59]. With the further increase in temperature, when the test temperature reached more than 300 °C, the thermal decomposition of phenolic resin binder led to a decrease in the degree of bonding between the components of the raw materials, resulting in the wear rate of the brake friction material specimens to increase more rapidly, with the wear rate of the specimens at 350 °C ranging from 0.394 × 10^−7^~0.617 × 10^−7^ cm^3^(N·m)^−1^.

Figure 12b illustrates that the specimens of brake friction material containing a higher amount of additional plant fibers generally had higher wear rates. Among the plant fiber-reinforced brake friction materials, F-6 had the lowest wear rate (1.68 × 10^−7^ cm^3^/(N·m)), which was lower than that of the specimen with the lowest wear rate in the preceding section [54].

#### 3.2.3. Friction Wear Mechanism

**Figure 13:**
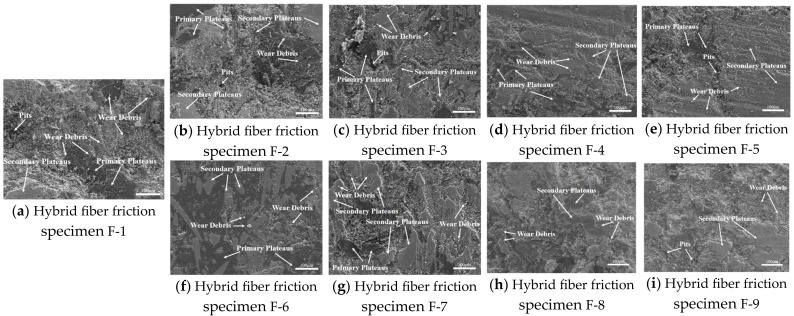
Wear surface micromorphology of 9 brake friction material specimens.

The wear surface micromorphology (SEM) of nine specimens of hybrid plant fiber-reinforced brake friction material is displayed in Figure 13. The wear surfaces of the F-1, F-2, F-3, F-7, and F-8 brake friction material specimens with higher fiber content were rougher, exhibiting more furrows, microcracks, and wear debris. These specimens also had secondary plateaus and showed broken fibers and pits, particularly on the F-1 and F-2 specimens. This is primarily due to the high content of plant fibers, which leads to the formation of hard particles, such as broken fibers, at the friction interface between the material matrix and the brake disc. These particles exacerbate both abrasive and adhesive wear of the brake friction material [54,56].

The wear surfaces of the brake friction material specimens of F-4, F-5, F-6, and F-9 were smoother compared to the previous five. This may be due to the plant fibers enhancing the bonding between the component raw materials in the formulation, thereby enhancing the friction and wear properties of the brake friction material [58,66]. Among them, the F-6 brake friction material specimen had the smoothest wear surface and the most secondary plateaus. This result is consistent with the wear performance test results, indicating that the F-6 material exhibited the best wear resistance and the highest degree of matrix bonding [41,51,61].

#### 3.2.4. Fuzzy Integrated Evaluation Method

In this experiment, the blurred comprehensive evaluation method was used to evaluate the friction factor and wear resistance of nine hybrid fiber-reinforced brake friction materials [22,67], and to determine their friction stability through the calculation process [68]. The specific methods are as follows:

Friction Factor Evaluation Method: The warming friction factor of the brake friction material at each test temperature is set to μf, and the cooling friction factor is set to μr, $\left|\mu f-\mu r\right|$. A larger value indicates that the brake friction material’s friction factor is more stable. For the evaluation of the friction factor, it is necessary to normalize $\left|\mu f-\mu r\right|$, after which a comparative analysis of the friction factor at each test temperature is carried out. Let the average friction factor fuzzy comprehensive evaluation value be $\Delta \mu^{*}$. The larger its value, the better the stability of the brake friction material. $\Delta \mu^{*}$ can be derived from Equations (6) and (7) [38].

(6) $$\Delta \mu^{*}=\frac{\sum_{i=1}^{5}\mathrm{Qi}\times \frac{\left(\left|\mu f-\mu r\right|\max-\left|\mu f-\mu r\right|i\right)}{\left(\left|\mu f-\mu r\right|\max-\left|\mu f-\mu r\right|\min\right)}+Q6}{n}$$

(7) $$Q=\left\{\begin{array}{c} 1.00,\mu \in \left[0.40,0.50\right]\\ 0.75,\mu \in \left[0.35,0.40\right)\cup \left(0.50,0.55\right]\\ 0.50,\mu \in \left[0.30,0.35\right)\cup \left(0.55,0.60\right]\\ 0.25,\mu \in \left[0.25,0.30\right)\cup \left(0.60,0.70\right]\\ 0.00,\mu \in \left[0.00,0.25\right)\cup \left(0.7,X\right] \end{array}\right.$$

where $\Delta \mu^{*}$ was the friction factor; μf was stability; μr was the warming friction factor at a certain test temperature; Qi was the cooling friction factor at a certain test temperature; μf and μr the greater weight within a certain test temperature; and i was the test temperature (100 °C, 150 °C, 200 °C, 250 °C, and 300 °C).

Wear rate evaluation method: Let $V^{*}$ denote each test temperature and the corresponding wear rate of the brake friction material. The maximum wear rate ratio, as per the national standard GB 5763-2008 [35], is calculated using Formula (8). A smaller value indicates a lower wear rate of the brake friction material. $V^{*}$ is normalized and calculated according to Formula (9).

(8) $$V^{*}=\frac{\sum_{j=1}^{6}\left(V\left(T\right)/S\left(T\right)\right)}{n}$$

(9) $$V^{**}=1-\frac{V^{*}-V^{*}\min}{V^{*}\max-V^{*}\min}$$

where $V^{*}$ was the wear rate of the brake friction material; $V\left(T\right)$ was the wear rate of the brake friction material when the test temperature was T; $S\left(T\right)$ was the maximum wear rate when the test temperature was T, as stipulated in the national standard GB 5763-2008 [35]; and n was the number of test temperatures (generally six).

Comprehensive performance evaluation method: In evaluating the friction and wear performance of brake friction materials, the friction factor plays a more important role in the safety, stability, and braking time of the braking process than the wear resistance. Therefore, the comprehensive performance F of brake friction materials is defined in this chapter’s tests and calculated using Formula (10).

(10) F=0.7μ*+0.3V**

From Table 5, it can be seen that the friction stability evaluation results of the nine specimens are in the following order from the largest to the smallest: F-6 > F-5 > F-4 > F-9 > F-8 > F-7 > F-3 > F-2 > F-1. The F-6 brake friction material specimen has the best friction stability. The wear evaluation results after the normalization process are in the following order from the largest to the smallest: F-6 > F-9 > F-5 > F-4 > F-8 > F-2 > F-1 > F-7 > F-3. The F-6 has the best wear resistance. According to the fuzzy comprehensive performance evaluation, the F-6 brake friction material specimen demonstrates excellent friction stability and wear resistance.

## 4. Conclusions

This paper investigates the feasibility of using coconut fiber and dypsis lutescens fiber for automotive-reinforced brake friction materials, focusing on environmental protection requirements and resource recycling, based on the design theory of brake friction materials. The effects of these two reinforcing fibers on the physical properties, friction and wear characteristics, and micromorphology of the worn surface of the friction material were compared and analyzed. The experimental results indicate that the addition of coconut fiber and dypsis lutescens fiber can significantly improve the physical properties and friction and wear properties of brake friction materials. When the fiber content is 6 wt.%, it maximizes the comprehensive performance of brake friction materials. Due to the effective reinforcement provided by the two fibers on the friction material, nine hybrid plant fiber-reinforced brake friction materials were designed by jointly using the golden section method and the orthogonal test method, and the effects of hybrid plant fibers on various properties and the micromorphology of the friction materials were further analyzed. The comprehensive tribological performance was evaluated using the fuzzy comprehensive evaluation method. Experimental results showed that the F-6 hybrid fiber-reinforced brake friction materials exhibit superior physical properties, thermal degradation resistance, recovery properties, and abrasion resistance compared to single-fiber-reinforced brake friction materials.

## Figures and Tables

**Table 1:** Raw material specifications and manufacturers.

Component Category	Material Name	Specification	Manufacturer
Binder	Phenolic resin	2123 Thermosetting	Mianzhuo, Zhengzhou, China
Reinforcement Fiber	Coconut fiber	Diameter 0.1 mm	Chuangying, Changzhou, China
	Dypsis lutescens fiber	Diameter 0.1 mm	Chuangying, Changzhou, China
	Composite mineral fiber	1~5 mm	Mayue, Shijiazhuang, China
	Sepiolite fiber	1~4 mm	Jiashuo, Shijiazhuang, China
Filler	Calcium carbonate	1250 Mesh	Yousuo, Linyi, China
	Vermiculite powder	20~40 Mesh	Xuyang, Shijiazhuang, China
	Precipitated barium sulfate	1250 Mesh	Yousuo, Linyi, China
Modifier	Flake graphite	99% High purity	Mianzhuo, Zhengzhou, China
	Petroleum coke	400 Mesh	Lipan, Shijiazhuang, China
	Aluminum oxide	600 Mesh	Mianzhuo, Zhengzhou, China
	Friction dust	40~100 Mesh	Mianzhuo, Zhengzhou, China
	Zinc stearate	13.5~15.5% (ZnO)	Yatai, Wuxi, China

**Table 2:** Formulation of coconut fiber-reinforced brake friction material.

Raw Materials (by wt.%)	Specimens				
	I-0	C-2	C-4	C-6	C-8
Coconut fiber	0	2.00	4.00	6.00	8.00
Sepiolite fiber	5.00	5.00	5.00	5.00	5.00
Compound mineral fibers	20.00	20.00	20.00	20.00	20.00
Phenolic resin	9.00	9.00	9.00	9.00	9.00
Flake graphite	8.00	8.00	8.00	8.00	8.00
Petroleum coke	7.00	7.00	7.00	7.00	7.00
Aluminum oxide	6.00	6.00	6.00	6.00	6.00
Friction dust	2.00	2.00	2.00	2.00	2.00
Calcium carbonate	13.00	13.00	13.00	13.00	13.00
Vermiculite powder	5.00	5.00	5.00	5.00	5.00
Precipitated barium sulfate	24.00	22.00	20.00	18.00	16.00
Zinc stearate	1.00	1.00	1.00	1.00	1.00

**Table 3:** Formulation of dypsis lutescens fiber-reinforced brake friction materials.

Raw Materials (by wt.%)	Specimens				
	I-0	S-2	S-4	S-6	S-8
Dypsis lutescens fiber	0	2.00	4.00	6.00	8.00
Sepiolite fiber	5.00	5.00	5.00	5.00	5.00
Compound mineral fibers	20.00	20.00	20.00	20.00	20.00
Phenolic resin	9.00	9.00	9.00	9.00	9.00
Flake graphite	8.00	8.00	8.00	8.00	8.00
Petroleum coke	7.00	7.00	7.00	7.00	7.00
Aluminum oxide	6.00	6.00	6.00	6.00	6.00
Friction dust	2.00	2.00	2.00	2.00	2.00
Calcium carbonate	13.00	13.00	13.00	13.00	13.00
Vermiculite powder	5.00	5.00	5.00	5.00	5.00
Precipitated barium sulfate	24.00	22.00	20.00	18.00	16.00
Zinc stearate	1.00	1.00	1.00	1.00	1.00

**Table 4:** Nine hybrid fiber brake friction material formulations.

	F-1	F-2	F-3	F-4	F-5	F-6	F-7	F-8	F-9

Coconut fiber	20.00	20.00	20.00	7.64	7.64	7.64	12.36	12.36	12.36
Dypsis lutescens fiber	20.00	12.36	7.64	20.00	12.36	7.64	20.00	12.36	7.64
Compound mineral fibers	20.00	12.36	7.64	7.64	20.00	12.36	12.36	7.64	20.00
Precipitated barium sulfate	1.21	1.67	1.96	1.96	1.82	1.92	1.68	2.05	1.82
Calcium carbonate	7.88	10.89	12.75	12.75	11.82	12.48	10.89	13.32	11.82
Vermiculite powder	4.85	6.70	7.84	7.85	7.27	7.68	6.70	8.20	7.27
Flake graphite	5.45	7.54	8.83	8.83	8.18	8.64	7.54	9.22	8.18
Petroleum coke	4.24	5.86	6.87	6.86	6.36	6.72	5.86	7.17	6.36
Aluminum oxide	2.42	3.35	3.92	3.92	3.64	3.84	3.35	4.10	3.64
Friction dust	3.64	5.03	5.88	5.88	5.45	5.76	5.02	6.15	5.45
Zinc stearate	1.82	2.51	2.94	2.94	2.73	2.88	2.51	3.08	2.73
Phenolic resin	8.49	11.73	13.73	13.73	12.73	13.44	11.73	14.35	12.73

**Table 5:** Fuzzy comprehensive evaluation results of 9 specimens.

	F-1	F-2	F-3	F-4	F-5	F-6	F-7	F-8	F-9
μ*	0.29	0.32	0.35	0.41	0.44	0.47	0.37	0.38	0.40
V**	0.52	0.58	0.00	0.76	0.82	1.00	0.47	0.62	0.89
F	0.359	0.398	0.245	0.515	0.554	0.629	0.40	0.452	0.547

## Data Availability

Data are contained within the article.
